# Supplementary material for: Lysophosphatidic Acid Receptor 5 (LPA5) Knockout Ameliorates the Neuroinflammatory Response In Vivo and Modifies the Inflammatory and Metabolic Landscape of Primary Microglia In Vitro
Source: Cells. 2022 Mar 22;11(7):1071. doi: 10.3390/cells11071071 (PMC8998093; doi:10.3390/cells11071071)
Supplement: Supplementary file 1 [file cells-11-01071-s001.zip › cells-1632817-supplementary.pdf]

Supplementary Information

Lysophosphatidic acid receptor 5 (LPA_5) knockout ameliorates the neuroinflammatory response in vivo and modifies the inflammatory and metabolic landscape of primary microglia in vitro

Lisha Joshi[1], Ioanna Plastira[1], Eva Bernhart[1], Helga Reicher[1], Zhanat Koshenov[1], Wolfgang F. Graier[1,2], Nemanja Vujic[1], Dagmar Kratky[1], Richard Rivera[3], Jerold Chun[3], Wolfgang Sattler[1,2]

[1] Division of Molecular Biology and Biochemistry, Gottfried Schatz Research Center, Medical University of Graz, 8010 Graz, Austria (lisha.joshi@medunigraz.at, ioanna.plastira@medunigraz.at, eva.bernhart@medunigraz.at, helga.reicher@medunigraz.at, zhanat.kostenov@medunigraz.at, wolfgang.graier@medunigraz.at, nemanja.vujic@medunigraz.at, dagmar.kratky@medunigraz.at, wolfgang.sattler@medunigraz.at)
[2] BioTechMed-Graz, 8010 Graz, Austria (wolfgang.graier@medunigraz.at, dagmar.kratky@medunigraz.at, wolfgang.sattler@medunigraz.at)
[3] Translational Neuroscience Initiative, Sanford Burnham Prebys Medical Discovery Institute, La Jolla, CA, USA (rrivera@sbpdiscovery.org, jchun@sbpdiscovery.org)

* Correspondence: wolfgang.sattler@medunigraz.at; Tel.: +43-316-385-71950

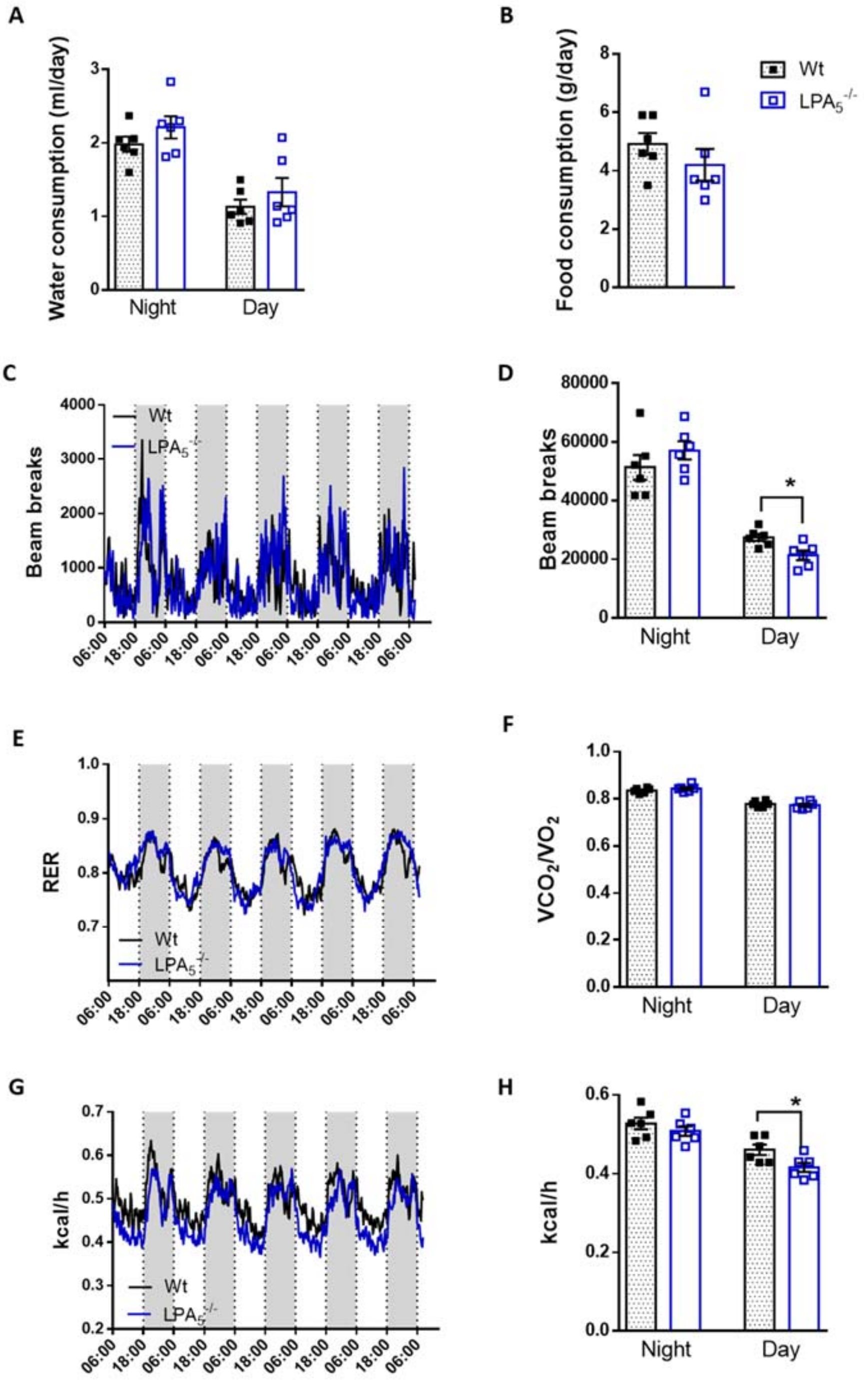

Supplementary Figure S1: In vivo energy metabolism during the 5d adaptation phase (without LPS treatment). Wt and $LPA_5^{-/-}$ mice were housed at room temperature in metabolic cages with free access to chow diet and water. The following parameters were measured/calculated: (**A**) Water consumption, (**B**) food intake, (**C**) real time locomotor activity, (**D**) mean locomotor activity, (**E**) real time measurement of respiratory exchange ratio (RER), (**F**) mean respiratory RER, (**G**) real time energy expenditure (EE) measurement, and (**H**) mean EE. (Data represent means (n = 6) ± SEM for wt and $LPA_5^{-/-}$ mice. Significance was calculated by Student`s t-test. * $p < 0.05$ compared to wt.

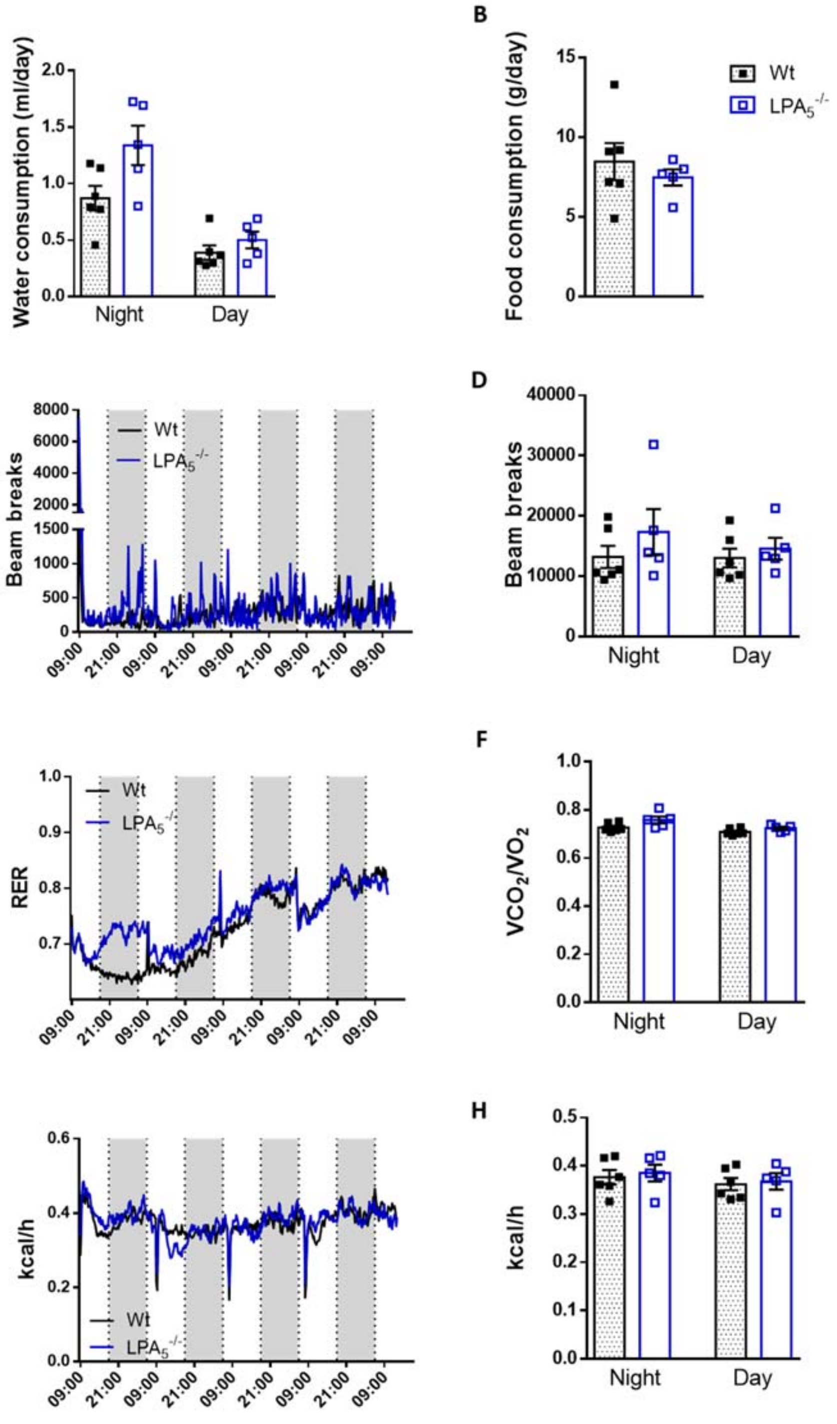

Supplementary Figure S2: Slightly improved metabolic performance of LPA$_5^{-/-}$ mice after long-term LPS treatment. Wt and LPA$_5^{-/-}$ mice were housed at room temperature in metabolic cages with free access to chow diet and water. Mice were injected daily with LPS (i.p, 1.4 mg/kg body weight) for 4 d. (**A**) Water consumption, (**B**) food intake, (**C**) real time locomotor activity, (**D**) mean locomotor activity, (**E**) real time measurement of respiratory exchange ratio (RER), (**F**) mean respiratory RER, (**G**) real time energy expenditure (EE) measurement, and (**H**) mean EE. (Data represent means ± SEM for wt (n = 6) and LPA$_5^{-/-}$ (n = 5) mice (due to bad general condition one mouse had to be euthanized during the first 24 h after the first LPS injection). Significance was calculated by Student`s t-test.

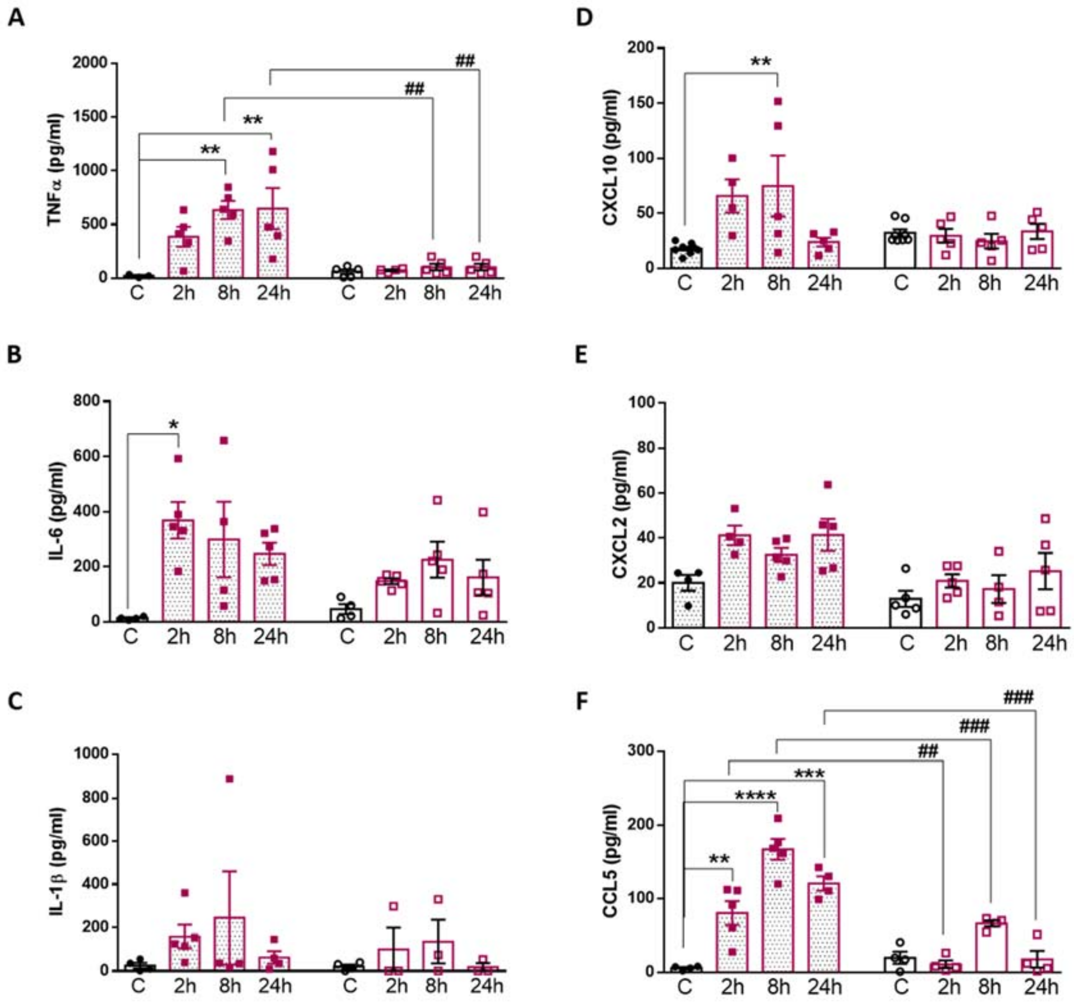

Supplementary Figure S3: LPA_5 regulates LPA-induced secretion of cyto-/chemokines in primary microglia. Wt and $LPA_5^{-/-}$ microglia were treated in the absence ('C') or presence of LPA (5 μM). At the indicated time points, supernatants were collected and (**A-C**) cytokine and (**D-F**) chemokine concentrations were quantified by ELISA. Values are expressed as mean ± SEM of five independent experiments. (*p < 0.05, ***p < 0.001, **** p < 0.0001 compared to wt control; ## p < 0.01, ### p < 0.001 $LPA_5^{-/-}$ condition compared to LPA-treated wt cells (two-way ANOVA with Bonferroni correction).

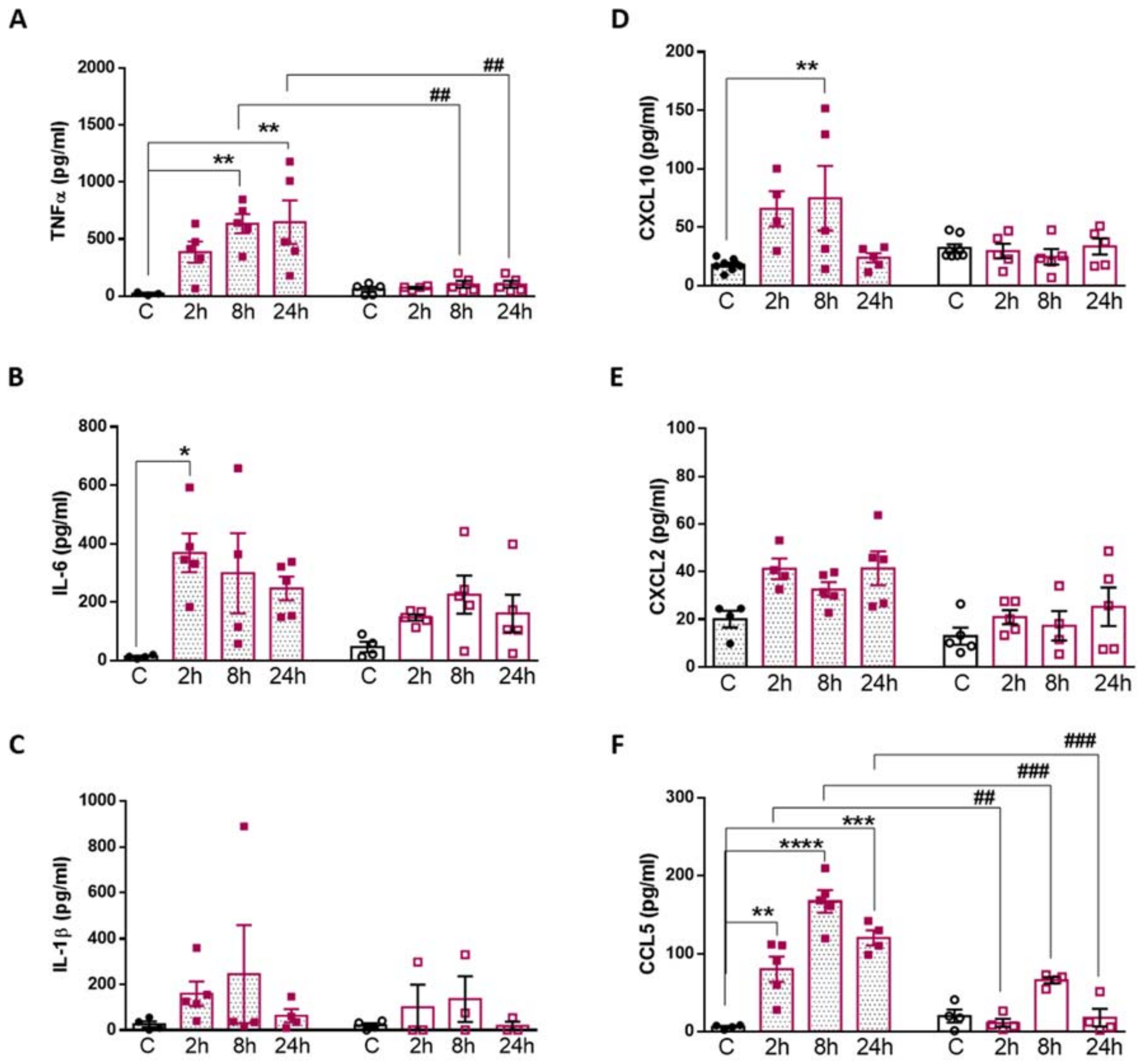

Supplementary Figure S4: LPA5 regulates mitochondrial respiration in primary microglia. (**A-B**) Oxygen consumption rate (OCR) in the absence and presence of LPA (5 μM) for 2h and 24h was measured using the XF Cell Mito Stress Test. Microglia isolated from wt and $LPA_5^{-/-}$ mice were treated with 2 μM oligomycin, 1.75 μM FCCP, and 2.5 μM antimycin A in XF assay medium to assess mitochondrial function. Bar graphs show (**C**) basal mitochondrial respiration, (**D**) maximal mitochondrial respiration, (**E**) ATP linked respiration, and (**F**) spare respiratory capacity. Values are expressed as mean ± SEM of three independent experiments (# $p < 0.05$, ### $p < 0.001$ $LPA_5^{-/-}$ compared to wt cells; two-way ANOVA with Bonferroni correction)

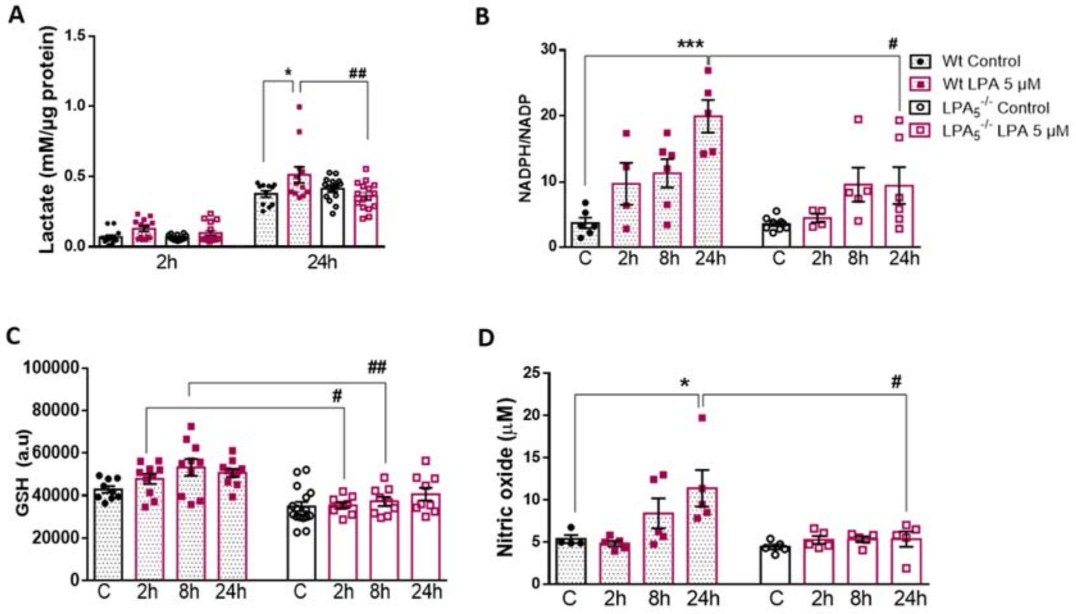

Supplementary Figure S5: LPA$_5$ regulates extracellular lactate content, NADPH/NADP ratio, glutathione concentration, and NO production in primary microglia. Wt and LPA$_5^{-/-}$ cells were cultivated in serum-free medium in the absence or presence of LPA (5 µM). (**A**) Lactate release by wt and LPA$_5^{-/-}$ cells was measured by EnzyChrom™ Glycolysis Assay Kit and compared to their appropriate controls. (**B**) Serum-starved cells were incubated in the absence or presence of LPA for the indicated times. NADPH/NADP ratio of the cells was measured by NADP/NADPH assay kit (Abcam), and compared to their control. (**C**) Glutathione (GSH) concentration of LPA treated wt and LPA$_5^{-/-}$ cells was quantified with GSH-Glo Assay kit and compared with controls. (**D**) NO production was determined by measuring the total nitrate concentration in the supernatants. Results are presented as mean values ± SEM of three independent experiments (* $p < 0.05$, *** $p < 0.001$ compared to control ('C'; # $p< 0.05$, ## $p < 0.01$ LPA$_5^{-/-}$ compared to wt cells, two-way ANOVA with Bonferroni correction
